# Vitamin D_3_ Metabolic Enzymes in the Porcine Uterus: Expression, Localization and Autoregulation by 1,25(OH)_2_D_3_ In Vitro

**DOI:** 10.3390/ijms23073972

**Published:** 2022-04-02

**Authors:** Malgorzata Grzesiak, Kinga Kaminska, Aleksandra Bodzioch, Ewa M. Drzewiecka, Anita Franczak, Katarzyna Knapczyk-Stwora

**Affiliations:** 1 Department of Endocrinology, Institute of Zoology and Biomedical Research, Jagiellonian University in Krakow, Gronostajowa 9, 30-387 Krakow, Poland; kinga.kaminska@doctoral.uj.edu.pl (K.K.); olaa09@vp.pl (A.B.); katarzyna.knapczyk@uj.edu.pl (K.K.-S.); 2 Department of Animal Anatomy and Physiology, Faculty of Biology and Biotechnology, University of Warmia and Mazury in Olsztyn, Oczapowskiego 1A, 10-718 Olsztyn, Poland; ewa.drzewiecka@uwm.edu.pl (E.M.D.); anitaf@uwm.edu.pl (A.F.)

**Keywords:** CYP27B1, CYP24A1, vitamin D_3_, uterus, pig

## Abstract

The role of vitamin D_3_ has been confirmed in female reproductive organs. This study aimed to examine vitamin D_3_ metabolic enzymes, i.e., CYP27B1 and CYP24A1, mRNA transcript and protein abundance, and protein localization in the uterus of pigs on days 2–5, 10–12, 15–16 and 18–20 of the estrous cycle. Additionally, we determined 1,25(OH)_2_D_3_ concentration in uterine flushings and the effect of 1,25(OH)_2_D_3_ (10, 50 and 100 ng/mL) in vitro on *CYP27B1* and *CYP24A1* mRNA transcript abundance in endometrial and myometrial slices. In the endometrium, a greater *CYP27B1* mRNA transcript abundance was noted on days 10–12 and 18–20 than on days 15–16, whereas encoded protein abundance was greater on days 18–20 when compared to days 15–16. Endometrial *CYP24A1* mRNA transcript abundance was greater on days 18–20 than on days 10–12 and 15–16. In the myometrium, *CYP27B1* mRNA transcript abundance was greater on days 18–20 than on days 2–5 and 15–16, while protein abundance was larger in slices collected on days 18–20 than on days 15–16. Neither *CYP24A1* mRNA transcript nor encoded protein abundance were detected in the myometrium. The highest 1,25(OH)_2_D_3_ concentration in uterine flushings was observed on days 18–20. Furthermore, the 1,25(OH)_2_D_3_ increased the abundance of the *CYP24A1* mRNA transcript in endometrial slices. Overall, our results suggest that porcine uterus is an extra-renal site of vitamin D_3_ metabolism. Both the endometrium and the myometrium possess the ability to synthesize vitamin D_3_, while only the endometrium contributes to its catabolism.

## 1. Introduction

In recent years, a broad role of vitamin D_3_ has been confirmed in various mammalian extra-skeletal tissues, including female reproductive organs such as the ovary, uterus, oviduct and placenta [1,2,3]. Given that vitamin D_3_ nuclear and membranous receptors, as well as metabolic molecules, are expressed in tissues of reproductive organs, vitamin D_3_ can regulate female reproductive function [4]. Indeed, vitamin D_3_ receptor (VDR)- and 1α-hydroxylase-deficient mice were infertile, exhibited uterine hypoplasia and impaired folliculogenesis [5,6]. In addition, disrupted vitamin D_3_ metabolism was described in reproductive pathologies such as ovarian cysts, endometriosis and uterine leiomyoma [7,8,9,10].

Vitamin D_3_ is purchased either from a diet or endogenous production in the skin [11]. The first step of vitamin D_3_ synthesis includes the conversion of 7-dehydrocholesterol to pre-vitamin D_3_ in keratinocytes under ultraviolet B irradiation, followed by thermal isomerization to cholecalciferol [11]. Further vitamin D_3_ bioactivation involves two hydroxylations; first, in the liver at position 25 to produce 25OHD_3_ (calcidiol) by 25-hydroxylases, and second, in the kidney at position 1 to generate 1,25(OH)_2_D_3_ (calcitriol) upon 1α-hydroxylase (CYP27B1) [12]. Both circulating metabolites, i.e., 25OHD_3_ and 1,25(OH)_2_D_3_, may be inactivated through 24-hydroxylase (CYP24A1) [13]. Of note, recent studies provided evidence of the novel pathway of vitamin D_3_ metabolism, which is dependent on CYP11A1 activation [14,15,16].

The critical steps in vitamin D_3_ metabolism are catalyzed by two enzymes, CYP27B1 and CYP24A1 [12]. CYP27B1 is responsible for the hydroxylation of 25OHD_3_ to a hormonally active form, 1,25(OH)_2_D_3_, which takes place in the mitochondria of the nephron’s proximal tubules [13]. The expression of renal 1α-hydroxylase is tightly regulated by parathormone (PTH), fibroblast growth factor 23 (FGF23) and calcitriol itself. In detail, PTH stimulates, while FGF23 and calcitriol inhibit, CYP27B1 catalytic activity [12]. Since CYP27B1 was found in non-renal tissues, the differences in the regulatory system of CYP27B1 beyond kidney were also demonstrated [17,18]. To date, CYP27B1 was not examined in the uterus, but it seems to be crucial regarding the contribution of extra-renal tissues to circulating vitamin D_3_ concentration. Both calcidiol and calcitriol levels are controlled through CYP24A1 [13]. Their 24-hydroxylation leads to the production of 24,25(OH)_2_D_3_ and calcitroic acid, respectively [12,13]. Depending on the species, CYP24A1 may also exhibit 23-hydroxylase activity and catalyze the production of biologically active lactones [19]. In most animal tissues, CYP24A1 is strongly stimulated by 1,25(OH)_2_D_3_ due to the presence of two vitamin D response elements (VDREs) in the promoter, and it is recognized as a marker of cell responsiveness to vitamin D_3_ [20].

The results of our past study documented VDR mRNA transcript and protein abundance in the porcine uterus throughout the estrous cycle, and the presence of 25OHD in uterine flushings [21]. Furthermore, it was found that active vitamin D_3_ affected myometrial estradiol-17β release in vitro [21]. Notably, there is no research describing the local vitamin D_3_ metabolism in the uterus of pigs. To fulfill this knowledge gap, we hypothesized herein that the porcine uterus possesses the ability to metabolize vitamin D_3_ throughout the entire estrous cycle and 1,25(OH)_2_D_3_ affects *CYP27B1* and *CYP24A1* mRNA transcript abundance in uterine tissues. Accordingly, this study was designed to examine: (i) the concentration of 1,25(OH)_2_D_3_ in uterine flushings; (ii) CYP27B1 and CYP24A1 mRNA transcript and protein abundance, and proteins localization in the porcine endometrium and myometrium, collected on days 2–5, 10–12, 15–16 and 18–20 of the estrous cycle; as well as (iii) the effect of 1,25(OH)_2_D_3_ in vitro on *CYP27B1* and *CYP24A1* mRNA transcript abundance in the endometrium and the myometrium, collected on days 12–13 of the estrous cycle.

## 2. Results

### 2.1. Concentration of 1,25(OH)_2_D_3_ in Uterine Flushings

**Figure 1:**
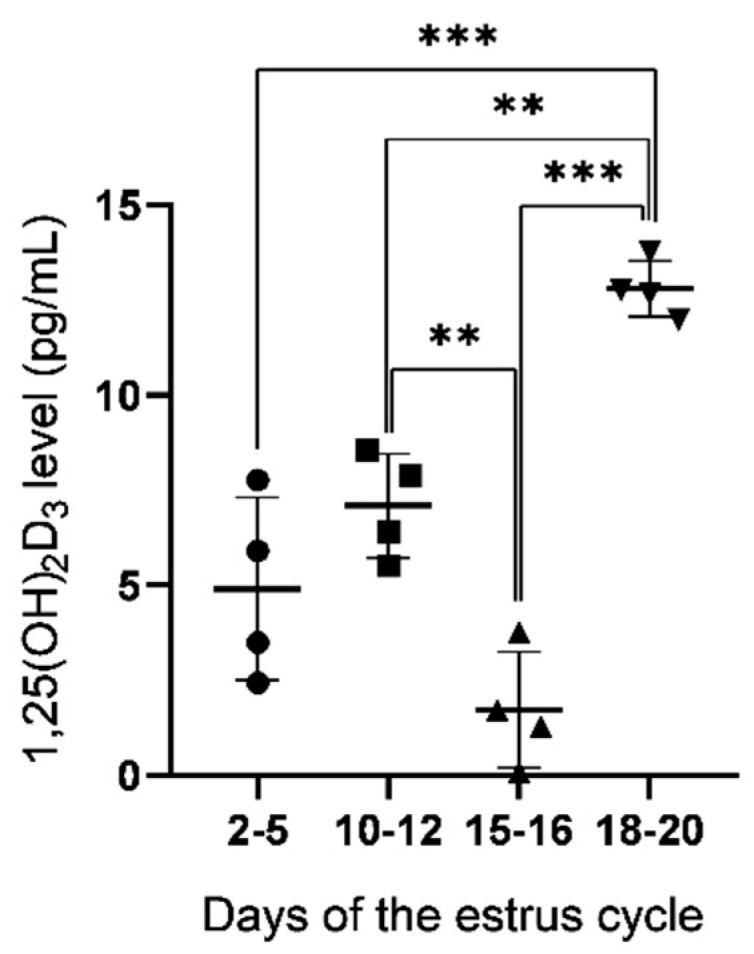
The concentration of 1,25(OH)_2_D_3_ (mean ± SD) in uterine flushings obtained from pigs on days 2–5, 10–12, 15–16 and 18–20 of the estrous cycle. ** *p* < 0.01; *** *p* < 0.001 (one-way ANOVA followed by Tukey *post hoc* test). *n* = 4 per each group.

The concentration of 1,25(OH)_2_D_3_ in uterine flushings collected from gilts on days 2–5, 10–12, 15–16 and 18–20 of the estrous cycle was measured using the ELISA method (Figure 1). The level of 1,25(OH)_2_D_3_ was the highest on days 18–20 with significant differences when compared to days 2–5 (*p* < 0.001), 10–12 (*p* < 0.01) and 15–16 (*p* < 0.001). A lower 1,25(OH)_2_D_3_ concentration was found on days 15–16 than on days 10–12 (*p* < 0.01) and 18–20 (*p* < 0.001) of the estrous cycle.

### 2.2. Abundance of CYP27B1 and CYP24A1 mRNA Transcripts in the Porcine Uterus

**Figure 2:**
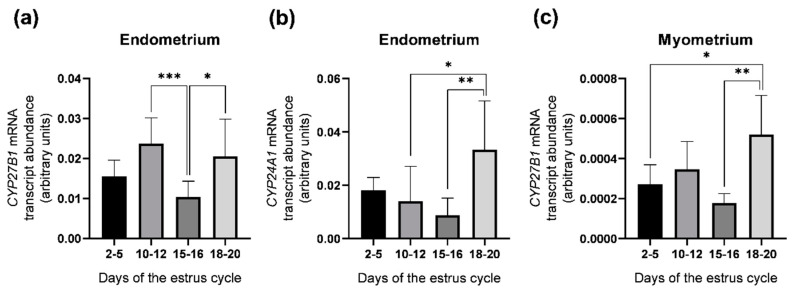
Relative mRNA transcript abundance of *CYP27B1* and *CYP24A1* in the endometrium (**a**,**b**, respectively) and *CYP27B1* in the myometrium (**c**) obtained from pigs on days 2–5, 10–12, 15–16 and 18–20 of the estrous cycle. Relative mRNA transcript abundance (quantitative real–time PCR) was expressed as the ratio relative to *GAPDH* (glyceraldehyde–3–phosphate dehydrogenase) and was presented as 2^−ΔCt^. Each value represents the mean ± SD. * *p* < 0.05; ** *p* < 0.01; *** *p* < 0.001 (one–way ANOVA followed by Tukey *post hoc* test). *n* = 5 per each group.

The abundance of *CYP27B1* and *CYP24A1* mRNA transcripts was examined in the porcine endometrial and myometrial slices obtained on days 2–5, 10–12, 15–16 and 18–20 of the estrous cycle by real–time PCR (Figure 2). In the endometrium, *CYP27B1* mRNA transcript abundance was greater on days 10–12 (*p* < 0.001) and 18–20 (*p* < 0.05) than on days 15–16 of the estrous cycle (Figure 2a), whereas *CYP24A1* mRNA transcript abundance was greater on days 18–20 in comparison to days 10–12 (*p* < 0.05) and 15–16 (*p* < 0.01) (Figure 2b). In the myometrium, only the abundance of the *CYP27B1* mRNA transcript, but not *CYP24A1*, was found and it was higher on days 18–20 when compared to days 2–5 (*p* < 0.05) and 15–16 (*p* < 0.01) of the estrous cycle (Figure 2c).

### 2.3. Abundance of CYP27B1 and CYP24A1 Proteins in the Porcine Uterus

**Figure 3:**
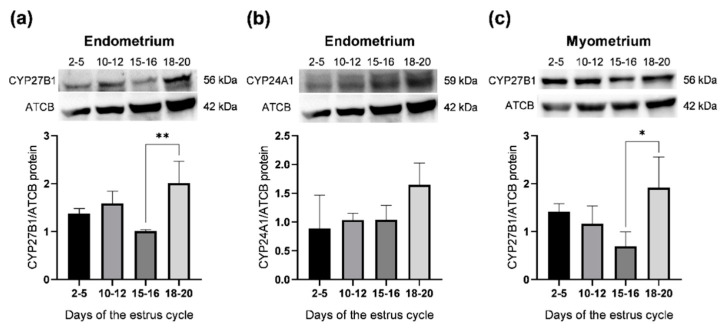
Relative protein abundance of CYP27B1 and CYP24A1 in the endometrium (**a**,**b**, respectively) and CYP27B1 in the myometrium (**c**) obtained from pigs on days 2–5, 10–12, 15–16 and 18–20 of the estrous cycle. The relative protein abundance was examined with densitometry and expressed as the ratio relative to β–actin (ACTB). Each value represents the mean ± SD. * *p* < 0.05; ** *p* < 0.01 (one–way ANOVA followed by Tukey *post hoc* test). *n* = 5 per each group.

The abundance of CYP27B1 and CYP24A1 proteins in the porcine endometrial and myometrial slices was examined on days 2–5, 10–12, 15–16 and 18–20 of the estrous cycle by a Western blot analysis (Figure 3). Antibodies recognized bands with predicted molecular weights of 56 and 59 kDa, respectively (Figure 3a–c, upper panels).

In the endometrium and the myometrium, the abundance of CYP27B1 proteins was greater on days 18-20 (*p* < 0.01 and *p* < 0.05, respectively) than in tissues collected on days 15–16 of the estrous cycle (Figure 3a,c, respectively). CYP24A1 protein was only detected in the endometrium and its abundance did not change throughout the entire estrous cycle (Figure 3b).

### 2.4. Localization of CYP27B1 and CYP24A1 in the Porcine Uterus

**Figure 4:**
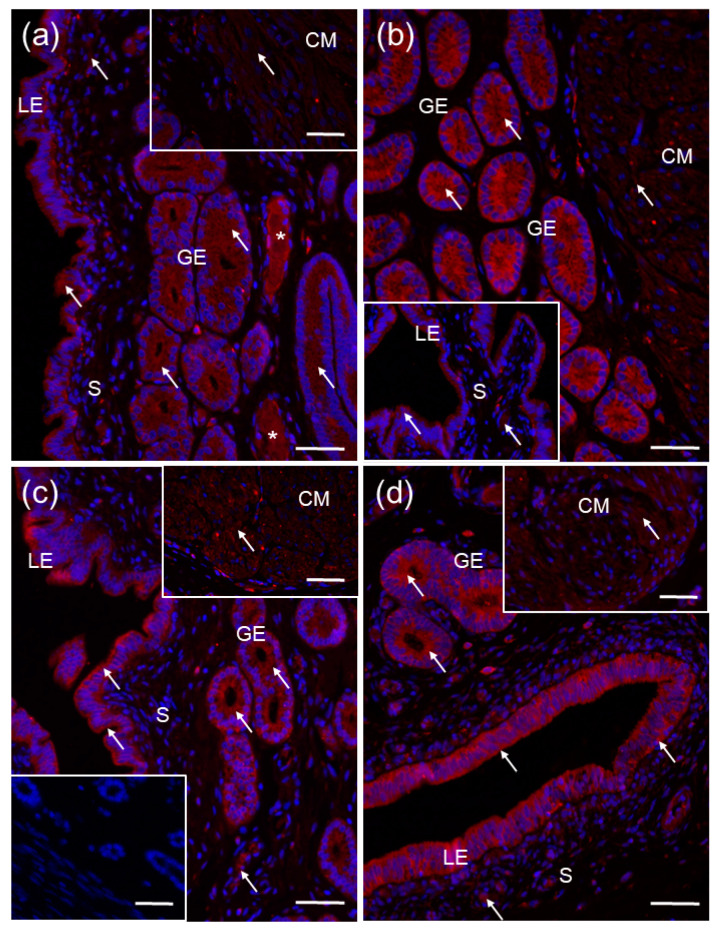
Representative micrographs of CYP27B1 immunofluorescent localization within porcine uterus on days 2–5 (**a**), 10–12 (**b**), 15–16 (**c**) and 18–20 (**d**) of the estrous cycle. Immunoreactive proteins were visualized using a Cy3 detection system (red). Nuclei were counterstained with DAPI (blue). Positive signal (*arrows*) was found in the cytoplasm of luminal (LE) and glandular (GE) epithelial cells of the endometrium, and myocytes within whole myometrial layer (only a circular myometrium (CM) is presented herein). Negative control (**c** lower inset) was obtained by the replacement of primary antibody by non–immune rabbit IgG. S, stroma; asterisks (*), blood vessels. Bar = 50 µm.

Positive red immunofluorescence for CYP27B1 was found in both the endometrium and the myometrium of the porcine uterus on days 2–5 (Figure 4a), 10–12 (Figure 4b), 15–16 (Figure 4c) and 18–20 (Figure 4d) of the estrous cycle. CYP27B1 was localized in the cytoplasm of luminal and glandular epithelial cells as well as some stroma cells (Figure 4a–d) and myocytes within circular and longitudinal myometrial layers (only circular myometrium is presented herein) (Figure 4a–d).

**Figure 5:**
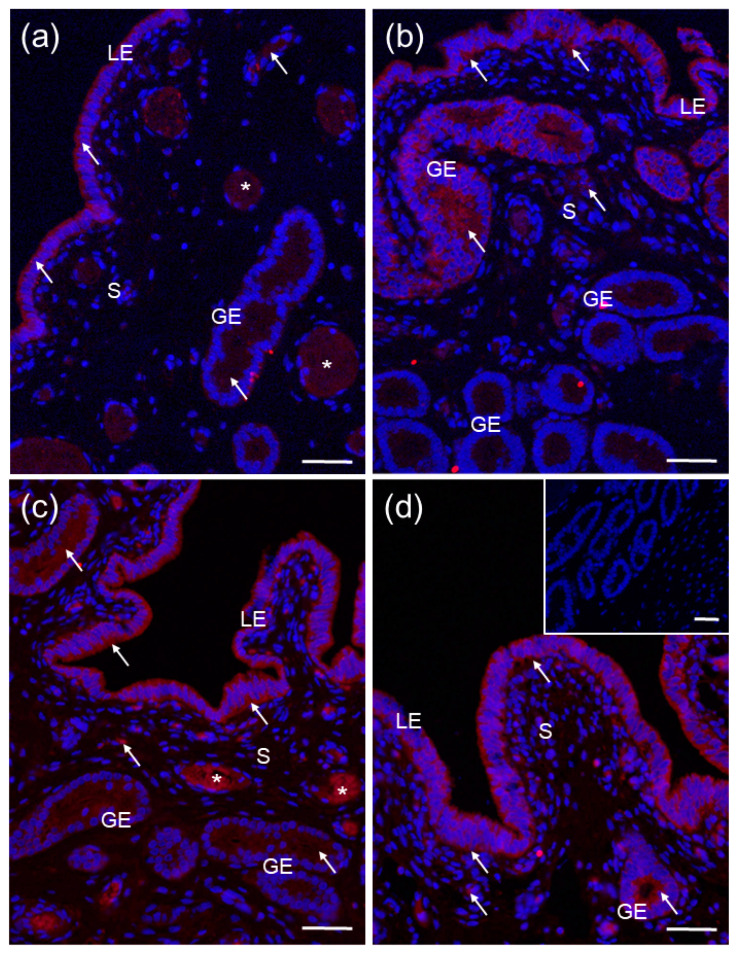
Representative micrographs of CYP24A1 immunofluorescent localization within porcine uterus on days 2–5 (**a**), 10–12 (**b**), 15–16 (**c**) and 18–20 (**d**) of the estrous cycle. Immunoreactive proteins were visualized using a Cy3 detection system (red). Nuclei were counterstained with DAPI (blue). Positive signal (*arrows*) was only found in the cytoplasm of luminal (LE) and glandular (GE) epithelial cells of the endometrium. Negative control (**d** inset) was obtained by the replacement of primary antibody by non–immune rabbit IgG. S, stroma; asterisks (*), blood vessels. Bar = 50 µm.

The CYP24A1 protein was found only in the porcine endometrium on days 2–5 (Figure 5a), 10–12 (Figure 5b), 15–16 (Figure 5c) and 18–20 (Figure 5d) of the estrous cycle, confirming the results from the Western blot analysis. A positive immunofluorescence was observed in the cytoplasm of luminal and glandular epithelial cells and some stroma cells (Figure 5a–d).

A positive signal for CYP27B1 (Figure 4a) and CYP24A1 (Figure 5a,c) was also found in blood vessels. There was no color reaction when sections were incubated with non–immune rabbit IgG (Figure 4c lower inset and Figure 5d inset) instead of a primary antibody.

### 2.5. Effect of 1,25(OH)_2_D_3_ on CYP27B1 and CYP24A1 mRNA Transcript Abundance in Endometrial and Myometrial Slices

**Figure 6:**
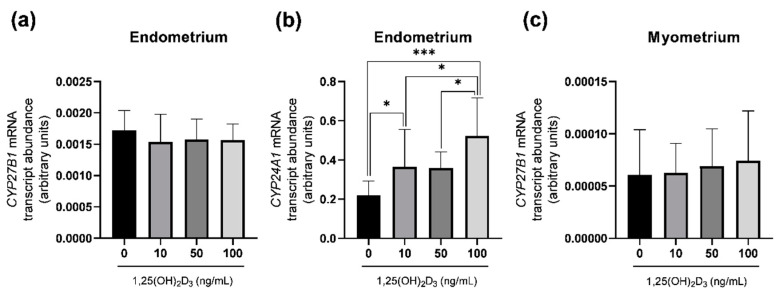
In vitro effect of 1,25(OH)_2_D_3_ (0, 10, 50 and 100 ng/mL) on *CYP27B1* and *CYP24A1* mRNA transcript abundance in endometrial slices (**a**,**b**, respectively), and *CYP27B1* mRNA transcript abundance in myometrial slices (**c**) harvested on days 12–13 of the estrous cycle. Relative mRNA transcript abundance (quantitative real–time PCR) is expressed as the ratio relative to *GAPDH* (glyceraldehyde–3–phosphate dehydrogenase) and is presented as 2^−ΔCt^. Each value represents the mean ± SD: * *p* < 0.05; *** *p* < 0.001 (one-way ANOVA followed by Tukey *post hoc* test); *n* = 5 per each group.

To examine the influence of active vitamin D_3_ in vitro on *CYP27B1* and *CYP24A1* mRNA transcript abundance in endometrial and myometrial slices of gilts, 1,25(OH)_2_D_3_ at doses 10, 50 and 100 ng/mL was applied (Figure 6).

1,25(OH)_2_D_3_ at 10 and 100 ng/mL (*p* < 0.05 and *p* < 0.001, respectively) significantly upregulated the abundance of the *CYP24A1* mRNA transcript in endometrial tissue in comparison to controls (Figure 6b). *CYP27B1* mRNA transcript abundance was unaffected by 1,25(OH)_2_D_3_ either in endometrial (Figure 6a) or myometrial (Figure 6b) slices.

## 3. Discussion

Recently, we have shown that porcine uterus expresses VDR; therefore, it is a target tissue for vitamin D_3_ [21]. The current study was undertaken to extend these results and verify the hypothesis of whether porcine uterus possesses the ability to metabolize vitamin D_3_ during the entire estrous cycle due to the presence of vitamin D_3_ metabolic molecules. Depending on the day of the estrous cycle, we showed CYP27B1 mRNA transcript and protein abundance, and protein localization in the porcine endometrium and myometrium, while the CYP24A1 mRNA transcript and protein were found only in the uterine endometrium. Apart from that we detected the 1,25(OH)_2_D_3_ in uterine flushings throughout the estrous cycle and further noted that calcitriol increased *CYP24A1* mRNA transcript abundance in endometrial slices in vitro.

The expression of vitamin D_3_ activating and inactivating enzymes was confirmed in various female reproductive organs, indicating that reproductive tissues might be an important site of vitamin D_3_ metabolism beyond the kidneys [4,17]. In the current study, we demonstrated CYP27B1 mRNA transcript and protein abundance in the porcine endometrium and myometrium throughout the estrous cycle. Using immunofluorescence, the CYP27B1 protein was detected in the cytoplasm of luminal and glandular epithelial cells, and stroma cells within the endometrium, as well as in myocytes. Our results are in agreement with previous research conducted on human cycling and pregnant endometrium [22,23], human myometrium [8] and pregnant porcine endometrium [24]. Moreover, the CYP24A1 mRNA transcript and protein were exclusively detected in the porcine endometrium, and its immunofluorescent localization reflected the aforementioned pattern of CYP27B1 distribution. At this time, research by Vigano et al. [22] showed *CYP24A1* mRNA transcript abundance in human endometrium, but protein abundance and tissue distribution have not yet been determined. As observed herein, the lack of vitamin D_3_ catabolic enzyme in the porcine myometrium is inconsistent with currently available results for the human myometrium, showing a low level of *CYP24A1* mRNA transcript abundance in normal myometrium and its overexpression in uterine leiomyoma [8]. It should be stressed that the myometrium of pigs has not been previously examined in the context of vitamin D_3_ metabolism and the revealed discrepancies might be species-specific. Taken together, our results implicate that, in pigs, both the endometrium and the myometrium are able to synthesize active vitamin D_3_ due to the expression of CYP27B1, while only the endometrium expresses the catabolizing enzyme, CYP24A1.

The present research demonstrates variations in the abundance of mRNA transcript and protein for vitamin D_3_–metabolizing enzymes within uterine compartments, depending on the studied days of the cycle. In the endometrium, a greater *CYP27B1* mRNA transcript abundance was noted on days 10–12 and 18–20 than on days 15–16, whereas protein was more abundant on days 18–20 in comparison to days 15–16 of the estrous cycle. Contrastingly, no significant variation in the expression of CYP27B1 was found in the human endometrium during the menstrual cycle [22,23]. Furthermore, herein we observed that the myometrial CYP27B1 mRNA transcript and protein abundances were greater on days 18–20 than on days 15–16 of the cycle. The high CYP27B1 level in the uterus in the follicular phase and low CYP27B1 expression in late luteal phase correspond to elevated and diminished 1,25(OH)_2_D_3_ concentrations in uterine flushings noted in these periods, respectively. According to research showing the release of 1,25(OH)_2_D_3_ by human endometrial cells in vitro [22], and detecting calcitriol in human myometrial tissue [8], it is likely that both uterus layers contribute to 1,25(OH)_2_D_3_ concentration in uterine microenvironment in pigs. Given that 1,25(OH)_2_D_3_ induced cell proliferation in goat granulosa cells [25] as well as in rat endometrial cell line [26], we further propose the possible role of a high intrauterine 1,25(OH)_2_D_3_ level within the follicular phase of the estrous cycle in the repairment of the porcine endometrium by modulation of cell proliferation and differentiation [27].

Apart from findings regarding CYP27B1, we showed changes in endometrial CYP24A1 abundance only at the transcript level; *CYP24A1* mRNA transcript abundance was higher on days 18–20 than on days 10–12 and 15–16 of the estrous cycle. CYP24A1 was shown to be directly regulated by 1,25(OH)_2_D_3_ due to the presence of VDRE in gene promoter [20,28]. Thus, we assumed that the endometrial abundance of the *CYP24A1* mRNA transcript was caused by an intrauterine calcitriol concentration that might serve as a local negative feedback mechanism. Despite the lack of variation in CYP24A1 protein abundance, we are not able to unequivocally state whether the local vitamin D_3_ inactivation in the endometrium contributes to 1,25(OH)_2_D_3_ concentration in uterine flushings in pigs.

Our current findings, showing the presence of vitamin D_3_ metabolic enzymes in the porcine uterus, prompted us to undertake research revealing whether 1,25(OH)_2_D_3_ regulated *CYP27B1* and *CYP24A1* mRNA transcript abundances in that tissue. In the in vitro experiment, 1,25(OH)_2_D_3_ upregulated *CYP24A1* mRNA transcript abundance in endometrial explants, but did not influence *CYP27B1* mRNA transcript abundance either in the endometrium or the myometrium of pigs. This part of the above mentioned results obtained for endometrium is consistent with data presented by Jang et al. [24], who also showed an increased *CYP24A1* gene transcription after 1,25(OH)_2_D_3_ treatment and unchanged *CYP27B1* mRNA transcript abundance in porcine endometrial explants. To date, this is the first study describing the in vitro effect of calcitriol on mRNA transcript abundance of vitamin D_3_–metabolic enzymes in the porcine myometrium. Regarding CYP24A1 regulation by 1,25(OH)_2_D_3_ in extra-renal tissues, this enzyme is strongly induced by calcitriol in most cells showing its expression, i.a., in keratinocytes [29] and syncytiotrophoblast cells [30]. The calcitriol-driven induction of *CYP24A1* transcription results from the presence of two VDREs in the promoter region [20]. Additionally, calcitriol can also enhance *CYP24A1* mRNA transcript abundance by recruiting histone H4 acetyltransferases and RNA polymerase II [31]. It is known that 1,25(OH)_2_D_3_ downregulates *CYP27B1* mRNA transcript abundance in the kidney; however, research has so far demonstrated different pathways of its regulation in non-renal tissues [12,17]. In keratinocytes and immune cells, calcitriol did not directly inhibit CYP27B1 [29,32]. On the other hand, in the placenta, 1,25(OH)_2_D_3_ inhibited *CYP27B1* transcription through VDR- and the cAMP-dependent mechanism [30]. Overall, the current research provides evidence of the direct regulation of endometrial *CYP24A1* mRNA transcript abundance by 1,25(OH)_2_D_3_, with no effect on *CYP27B1* in both the endometrium and the myometrium of pigs.

## 4. Materials and Methods

### 4.1. Animals and Sample Collection

The use of animals was in accordance with the Act of 15 of January 2015 on the Protection of Animals Used for Scientific or Educational Purposes and Directive 2010/63/EU of the European Parliament and the Council of 22 of September 2010 on the protection of animals used for scientific purposes.

Porcine uteri were harvested from sexually mature crossbred gilts (Large White × Polish Landrace; 100–110 kg body weight) at a local slaughterhouse under veterinarian control and transported on ice to the laboratory within ~1 h. Tissues were collected on days 2–5 (early luteal phase; *n* = 5), 10–12 (mid luteal phase; *n* = 5), 15–16 (late luteal phase; *n* = 5) and 18–20 (follicular phase; *n* = 5) of the estrous cycle following the verification of the estrous cycle stage by ovarian morphology and corpus luteum quality [33]. The uteri were flushed with 20 mL of phosphate-buffered saline (PBS, pH 7.4), and flushings were stored at −20 °C for analysis of 1,25(OH)_2_D_3_ concentration. Uterine horns were longitudinally opened on the mesometrial surface. The perimetrium was careful scraped using a scalpel blade and fragments of the endometrium and the myometrium were collected with scissors [34]. To assess CYP27B1 and CYP24A1 mRNA transcript (real–time PCR) and protein abundances (Western blot), small endometrial and myometrial sections of the middle part of uterine horns were snap–frozen in liquid nitrogen. Fragments of the uterine wall containing the endometrium and myometrium were fixed in 10% neutral-buffered formalin for immunofluorescence labeling of CYP27B1 and CYP24A1 proteins. Thereafter, fixed tissues were dehydrated in an increasing gradient of ethanol, cleared in xylene and embedded in paraplast (Sigma-Aldrich, St. Louis, MO, USA).

### 4.2. Incubation of Endometrial and Myometrial Slices In Vitro

To examine the effect of 1,25(OH)_2_D_3_ on *CYP27B1* and *CYP24A1* mRNA transcript abundance in endometrial and myometrial slices in vitro, uteri (*n* = 5) were collected from gilts on days 12–13 of the estrous cycle and prepared as described above. Endometrial and myometrial slices (200–210 mg weight, 3 mm thick) were incubated separately in culture vials containing 2 mL of Medium 199 (Sigma-Aldrich) supplemented with 0.1% bovine serum albumin (BSA) fraction V (Carl Roth GmbH þ Co KG, Mühlburg, Karlsruhe, Germany) and 1% antibiotic-antimycotic solution (AAS; Sigma-Aldrich) as previously shown [21]. After 18 h of preincubation in a shaking water bath under an atmosphere of 95% O_2_ and 5% CO_2_ at 37 °C, culture medium was replaced with fresh medium, then incubated for 6 h in the presence of control medium and supplemented with 1,25(OH)_2_D_3_ (Sigma-Aldrich) at doses 10, 50 and 100 ng/mL [21]. Next, tissue samples were collected and snap-frozen for RNA isolation and real–time PCR analysis. Each treatment was conducted in duplicate and the experiment was carried out five times (*n* = 5).

### 4.3. Quantitative Real-Time PCR Analysis

Total RNA was extracted from frozen endometrial and myometrial samples with TRI Reagent solution (Ambion, Austin, TX, USA) following the manufacturer’s instructions. The quantity and quality of the RNA were assessed by determining the A260/A280 ratio using a NanoDrop™ Lite Spectrophotometer (Thermo Scientific, Wilmington, DE, USA), and RNA integrity was evaluated by electrophoresis on 1 % formaldehyde–agarose gel. High-Capacity cDNA Reverse Transcription Kit (Applied Biosystems, Foster City, CA, USA) was used to obtain total cDNA from 1 µg of RNA of each sample. Quantitative real–time PCR was conducted with TaqMan Gene Expression Master Mix (Applied Biosystems) and porcine–specific TaqMan Gene Expression Assays (Applied Biosystems) for *CYP27B1* (assay ID: Ss03391198_m1) and *CYP24A1* (assay ID: Ss03391412_m1) following manufacturers’ protocol [35]. Glyceraldehyde–3–phosphate dehydrogenase (*GAPDH*; assay ID: Ss03373286_u1) was employed as an endogenous control. Real-time PCR reactions were performed in duplicate with StepOne™ Real-Time PCR System (Applied Biosystems) according to the recommended cycling program (2 min at 50 °C, 10 min at 95 °C, 40 cycles of 15 s at 95 °C, and 1 min at 60 °C). The amplification of contaminating genomic DNA was checked by control experiments in which reverse transcriptase was omitted during the reverse transcription step. The amount of each target cDNA was normalized with respect to the *GAPDH* (ΔCt value) as previously described [35]. The relative *CYP27B1* and *CYP24A1* mRNA transcript abundance was presented as 2^−ΔCt^, and these values were used to calculate statistical differences.

### 4.4. Western Blot Analysis

Total protein extraction and Western blot analysis were conducted as previously described [10,35]. Samples were separated by 10% SDS–PAGE (Mini-Protean TGX Precast Gels; Bio-Rad Laboratories Inc., GmbH, Munchen, Germany) and electroblotted onto a PVDF membrane (Trans-Blot Turbo Mini 0.2 µm PVDF Transfer Packs; Bio-Rad Laboratories Inc.) using a semi–dry Trans-Blot Turbo Transfer System (Bio-Rad Laboratories Inc.). The blotted membranes were blocked for 1 h at room temperature (RT) in 5% non–fat dry milk containing 0.1% Tween20 followed by overnight incubation at 4 °C with primary antibodies and then with secondary horseradish peroxidase–conjugated antibody for 1.5 h at room temperature (Table 1). Proteins were detected by chemiluminescence and images were captured with a ChemiDocTM XRS+ System (Bio-Rad Laboratories Inc.). Each membrane was stripped and reprobed with anti–β–actin antibody followed by respective secondary antibody (Table 1). The bands were densitometrically quantified and normalized to their corresponding β–actin bands using the public domain ImageJ program v. 1.8.0 (National Institutes of Health, Bethesda, MD, USA). Primary anti–CYP27B1 and anti–CYP24A1 were validated for porcine tissues in our previous experiment [35].

### 4.5. Immunofluorescence

Immunofluorescence labeling was performed as previously described [36]. Briefly, unmasking procedure with microwave heating in 0.01 M citrate buffer (pH 6.0) and blocking of non-specific binding sites with 5% normal goat serum prior to incubation with anti–CYP27B1 and anti–CYP24A1 primary antibodies was performed (Table 1). After overnight incubation at 4 °C in a humidified chamber, the antigens were visualized using Cy3–cojugated secondary antibody for 1.5 h in the dark (Table 1). Finally, sections were mounted in Vectashield Antifade Mounting Medium with 4′,6–diamidino-2-phenylindole (DAPI; Vector Lab., Burlingame, CA, USA) and examined with epifluorescence microscope Nikon Eclipse Ni-U (Nikon, Tokyo, Japan) with corresponding software. Negative controls were prepared by section incubation with non–immune rabbit IgG (NI01, Calbiochem, Darmstadt, Germany) instead of primary antibodies.

### 4.6. Analysis of 1,25(OH)_2_D_3_ Concentration in Uterine Flushings

The concentration of 1,25(OH)_2_D_3_ in the porcine uterine flushings was determined using an enzyme–linked immunosorbent assay kit (1,25(OH)_2_ Vitamin D ELISA; cat no. KAP1921; DIAsourceImmunoAssays, Louvain-la-Neuve, Belgium) following the manufacturer’s recommendation. Assay sensitivity was 0.8 pg/mL with ranges of 0–180 pg/mL. Intra- and interassay coefficients of variation were 5.0% and 13.2%, respectively. All analyses were performed in duplicate.

### 4.7. Statistical Analysis

Statistical analysis was performed using GraphPad Software (La Jolla, CA, USA). To verify the normal distribution of data, the Shapiro–Wilk and Lilliefors tests were applied. Due to the normal distribution, one–way ANOVA followed by Tukey *post hoc* test was used. All data are presented as the overall mean ± standard deviation (SD), and differences were considered statistically significant at the 95% confidence level (*p* < 0.05).

## 5. Conclusions

**Figure 7:**
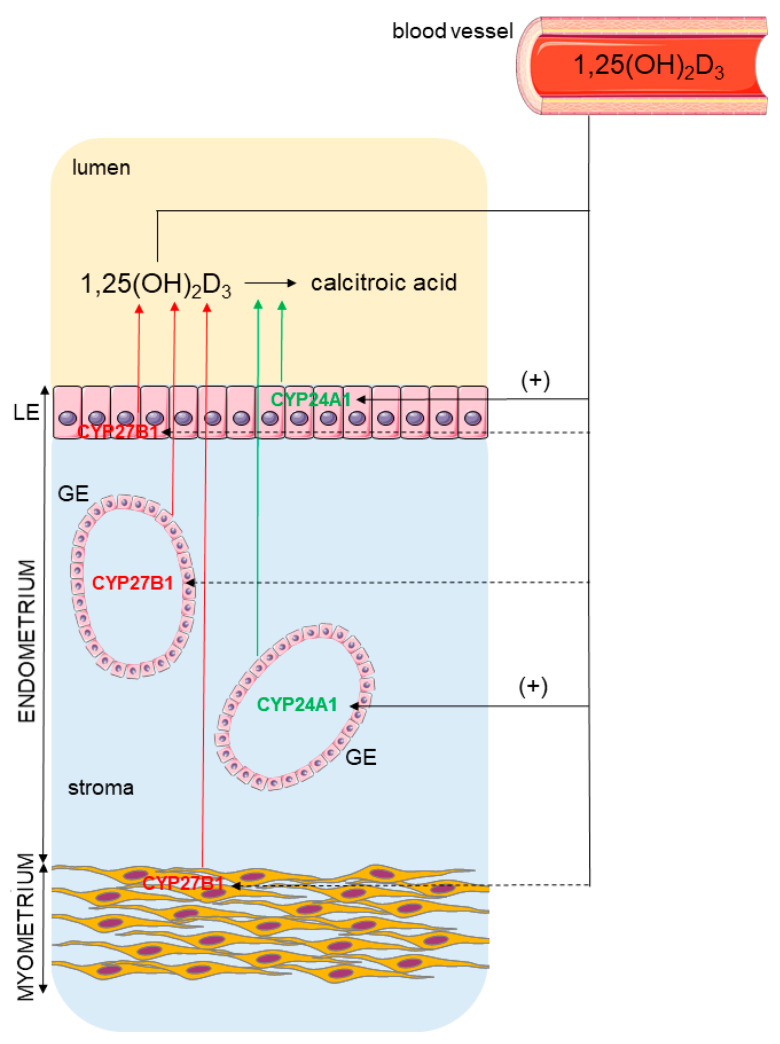
The pattern of CYP27B1 and CYP24A1 distribution in the porcine uterus and the proposed effect of 1,25(OH)_2_D_3_ on their abundance. The solid line indicates a positive effect (+), while the dashed line (---) indicates a lack of effect.

The present study demonstrates the capability of the porcine uterus to metabolize vitamin D_3_ within the course of the estrous cycle. In detail, the synthesizing enzyme, CYP27B1, was found in the porcine endometrium and myometrium, while the inactivating enzyme, CYP24A1, was detected only in the endometrial compartment. Furthermore, the presence of 1,25(OH)_2_D_3_ in uterine flushings contributes to our statement about the plausible intrauterine vitamin D_3_ metabolism (Figure 7). We further confirm the direct regulation of endometrial *CYP24A1* mRNA transcript abundance by 1,25(OH)_2_D_3_ in vitro, indicating the possible mechanism that might control calcitriol level in the uterus. Taken together, vitamin D_3_ could be considered as an important local regulator of uterine function in pigs.

## Figures and Tables

**Table 1:** Primary and secondary antibodies used for Western blot (WB) and immunofluorescence (IF).

Antibody	Serum	Host Species	Vendor	WB Dilution	IF Dilution
Anti–CYP27B1	5% NGS	Rabbit	Invitrogen, Carsband, CA, USA cat no. PA5-79128	1:3000	1:300
Anti–CYP24A1	5% NGS	Rabbit	Invitrogen, Carsband, CA, USA cat. no. PA5-79127	1:1000	1:300
Anti–β–actin	-	Mouse	Sigma-Aldrich, St. Louis, MO, USA cat. no. A2228	1:4000	-
Anti–rabbit IgG, Cy3	-	Goat	Thermo Fisher Scientific, DE, USA cat. no. A10520	-	1:100
Anti–rabbit IgG	-	Goat	Invitrogen, Carsband, CA, USA cat. no. 31460	1:3000	-
Anti–mouse IgG	-	Horse	Bio-Rad Laboratories Inc., GmbH, Munchen, Germany cat. no. 170-6516	1:3000	-

Abbreviations: CYP24A1, 24-hydroxylase; CYP27B1, 1α-hydroxylase; Cy3, cyanine3; NGS, normal goat serum.

## Data Availability

The raw data used for the preparation of the presented results are available on request from the corresponding author.
